# Neuromuscular System of Nematodes Is a Target of Synergistic Pharmacological Effects of Carvacrol and Geraniol

**DOI:** 10.3390/ph18081232

**Published:** 2025-08-20

**Authors:** Maja Stojković, Djordje S. Marjanović, Dragana Medić, Claude L. Charvet, Saša M. Trailović

**Affiliations:** 1 Department of Pharmacology, Clinical Pharmacology and Toxicology, Faculty of Medicine, University of Belgrade, 11000 Belgrade, Serbia; maja.stojkovic@med.bg.ac.rs; 2 Department of Pharmacology and Toxicology, Faculty of Veterinary Medicine, University of Belgrade, 11000 Belgrade, Serbia; marjanovicd@vet.bg.ac.rs (D.S.M.); dragana.medic@vet.bg.ac.rs (D.M.); 3 Institut National de Recherche pour l’Agriculture, l’Alimentation et l’Environnement (INRAE), Université de Tours, ISP, F-37380 Nouzilly, France; claude.charvet@msd.de

**Keywords:** geraniol, carvacrol, nAChR, Asu-ACR-16, *Caenorhabditis elegans*, *Ascaris suum*

## Abstract

**Background:** The active ingredients of essential plant oils appear as potentially effective antinematodal drugs or substances that can potentiate the action of already-existing anthelmintics. So far, we have verified that, aside from the direct effect on the neuromuscular system of nematodes, some of them can potentiate the effects of drugs that are agonists or antagonists of nematode cholinergic receptors. **Methods:** In this study, the antinematodal effects of geraniol and carvacrol were compared, as well as their interaction in the experimental model *Caenorhabditis elegans*, on the contractile properties of *Ascaris suum* neuromuscular preparations and on the ACR-16 nicotinic acetylcholine receptor (nAChR) of *A. suum* expressed in Xenopus leavis oocytes. **Results:** The combination of geraniol and carvacrol showed a synergistic nematocidal effect in the tests on *C. elegans*, reducing the value of individual LC_50_ by almost 10-times. This combination also exerted a synergistic inhibitory effect on the contractions of *A. suum*, significantly increased the EC_50_ of ACh and reduced the maximal contractile effect. The synergistic interaction of these two monoterpenes on Asu-ACR-16 nAChR expressed in Xenopus oocytes resulted in a significant decrease in the maximum current, while the ACh EC_50_ value remained unchanged. **Conclusions:** Our findings provide a better understanding of the mode of action of monoterpene plant compounds. The possible antiparasitic application of active ingredients of essential plant oils that exhibit a synergistic anthelmintic effect represents an important basis for the development of new drugs and new therapeutic procedures.

## 1. Introduction

Highly pathogenic nematode parasites pose a significant threat to humans and animals, causing widespread morbidity and significant socio-economic losses at the global level. Chemotherapy remains the mainstay for controlling all helminthiases, but there are at list two main problems that compromise the use of antinematodal drugs: increasing resistance of parasitic nematodes and the toxicity of drugs if their doses are increased. The neuromuscular system of parasitic nematodes has proven to be an efficient pharmacological target for anthelmintics [1]. Some of the most frequently used antiparasitic drugs are agonists of nicotinic acetylcholine receptors (nAChRs) (imidazothiazoles and tetrahydropyrimidines) or activators of both glutamate-gated chloride channels (GluCls) and GABA-receptors (macrocyclic lactones). Cholinergic agonists like levamisole, pyrantel, and oxantel work by specifically activating certain acetylcholine ion channels in the muscles of worms. This causes the muscles to contract strongly, which rapidly leads to the worms becoming paralyzed [2]. Plants naturally produce various compounds known as secondary metabolites, which have different pharmacological activities. From past studies on how these compounds act, it seems that essential oils and their active ingredients could be used effectively and safely as an alternative or in combination with standard antiparasitic medicines. Our research focuses on terpenoid-based active ingredients found in essential oils. This aligns with the worldwide effort to cut down on the use of synthetic drugs in animal care and move toward more plant-based treatments. On the other hand, considering the specific mechanism of the antinematodal action of AIs of EOs, this can be an effective way to neutralize parasitic helminths. Our previous results on plant monoterpenoid evidenced that the mechanism of antinematodal effects of carvacrol involved the inhibition of parasite muscle contraction. Specifically, carvacrol inhibited acetylcholine (ACh)-induced depolarizations of muscle cells, indicating direct interaction with nAChRs in *Ascaris suum* [3,4]. We observed that carvacrol enhanced the inhibitory effect of monepantel on *A. suum* contractions, which may have an effective clinical application. On the other hand carveol, another monoterpenoid potentiated the contractile effect of ACh in *A. suum*, indicating a significant platform for potentiating the antinematodal action of nicotinic acetylcholine receptor (nAChR) agonists [5]. It is obvious that the AIs of plant EOs possess anthelmintic potential that could be applied in the pharmacotherapy of parasitic infections in humans and animals.

Here, we decided to examine the properties of geraniol, a cyclic monoterpene alcohol that is used as a repellent [6,7]. Geraniol is the main ingredient of rose oil but is also present in many other EOs such as Palmarosa oil [8]. Geraniol was found to be the most effective constituent of Pelargonium graveolens EO against the parasitic root-knot nematode *Meloidogyne incognita* [9,10]. Another investigation assessing the anthelmintic activity of the Cymbopogon martinii EO on *Caenorhabditis elegans* resulted in geraniol as the anthelmintic component of palmarosa oil [11]. Geraniol also exhibited larvicidal activity against the genus of roundworms *Contracaecum* [12] and against marine nematodes *Anisakis simplex* [13]. Also, geraniol disrupts the hatching of eggs of different strains of *H. contortus* in vitro, with an EC_50_ value of 651.60 to 681.70 μM, as well as the development of larvae with an EC_50_ of 9.43 to 13.12 mM [14]. However, there are no data on the mechanism of the antinematodal actions of geraniol.

The aim of this study was to compare the antinematodal effects of geraniol and carvacrol, as well as their interaction in the model nematode *Caenorhabditis elegans*, as well as on the contractile model of the neuromuscular preparation *A. suum* and on the nicotinic acetylcholine receptor of *A. suum* ACR-16, expressed on Xenopus oocytes.

## 2. Results

### 2.1. Activity of Geraniol and Carvacrol on C. elegans

**Figure 1:**
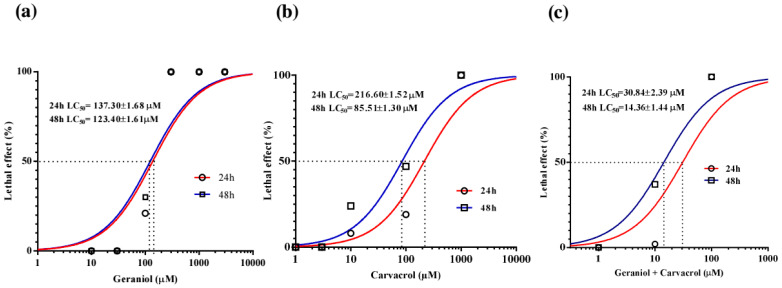
Lethal effect of increasing concentrations of geraniol (**a**), carvacrol (**b**) and their combination (**c**) on adult *C. elegans*.

The median lethal concentration (LC_50_) of geraniol for *C. elegans* after 24 h of exposure was 137.30 ± 1.68 μM and did not differ significantly after 48 h, although it decreased to 123.40 ± 1.61 μM (Figure 1a). Geraniol caused atonic paralysis of the nematodes, which occurred before pharyngeal pumping ceased. The LC_50_ value of carvacrol was 215.08 ± 1.18 μM after 24 h and 84 ± 1.13 μM after 48 h (Figure 1b). In the control experiments, no death of adult *C. elegans* individuals was recorded. Analyzing real-time motility recordings, it was observed that exposure to carvacrol leads to a slowing and cessation of pharyngeal pumping, which occurs before nematode movement ceases. When *C. elegans* was exposed to a combination of geraniol and carvacrol, the LC_50_ value after 24 h was 30.86 ± 2.39 μM. After 48 h of exposure, the LC_50_ value decreased twice to 14.36 ± 1.44 μM (Figure 1c).

### 2.2. Effect of Geraniol and Carvacrol on Ascaris suum Neuromuscular Contractions

**Figure 2:**
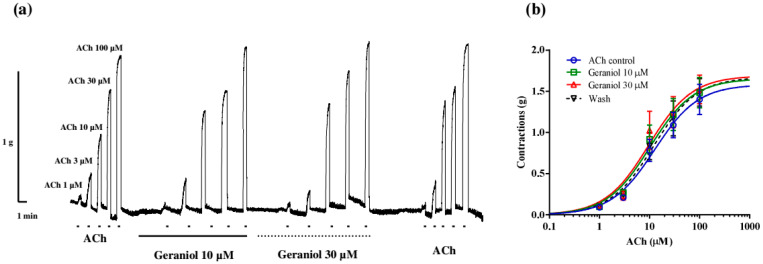
(**a**) Original recording of isometric contractions of *A. suum* muscle flap induced by increasing acetylcholine concentrations and the effect of geraniol (10 and 30 μM) on those contractions; (**b**) the concentration–response plot for acetylcholine control, in the presence of geraniol 10 and 30 μM and after washing (n = 6) (mean ± S.E.).

It was important for us to check whether geraniol affects the contractions of the neuromuscular preparation of *A. suum* induced by increasing concentrations of ACh (Figure 2a). In the control series of contractions, the measured EC_50_ value of ACh was 12.69 ± 1.40 µM, while in the presence of 10 µM of geraniol, it was reduced to 10.32 ± 1.43 µM, but the difference was not statistically significant (*p* = 0.65). Geraniol at a concentration of 30 µM reduced the EC_50_ of ACh to 9.60 ± 1.48 µM but also without statistical significance (*p* = 0.43). After washing, the EC_50_ value was 12.11 ± 1.44 µM (*p* = 0.99). Incubation of the preparation with geraniol did not lead to a statistically significant change in the maximal contractile effect (E_max_) of ACh. E_max_ in the control series of contractions was 1.58 ± 0.16 g, and in the presence of 10 and 30 µM of geraniol, 1.65 ± 0.0.17 g and 1.69 ± 0.19 g, respectively (*p* = 0.99 and 0.88). After washing and removing the geraniol from the bath solution, the E_max_ of contractions was 1.67 ± 0.19 g, which was not significantly different from the control value (*p* = 0.98) (Figure 2b).

**Figure 3:**
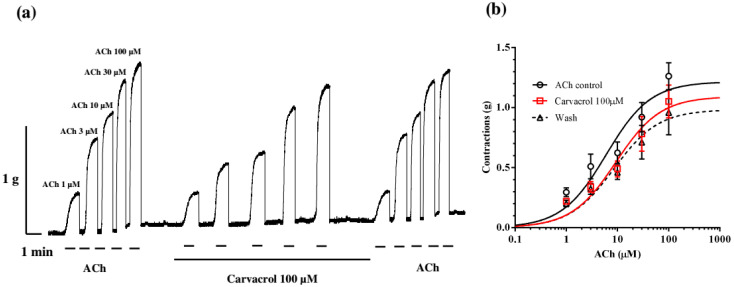
(**a**) Original recording of isometric contractions of *A. suum* muscle flap induced by increasing acetylcholine concentrations and the effect of carvacrol (100 μM) on those contractions; (**b**) the concentration–response plot for acetylcholine control, in the presence of carvacrol 100 μM and after washing (n = 6) (mean ± S.E.).

In a separate series of contractions, we examined the inhibitory effect of 100 μM of carvacrol on ACh-induced contractions. Increasing concentrations of ACh in the control series caused dose-dependent contractions of the *Ascaris suum* neuromuscular preparation, with an EC_50_ value of 6.03 ± 1.40 μM (Figure 3a). Carvacrol non-significantly increased the EC_50_ value of ACh to 9.35 ± 1.46 μM (*p* = 0.64), and it did not change even after removing carvacrol from the experimental bath, being 8.57 ± 0.20 μM (*p* = 0.28). Also, carvacrol non-significantly reduced the E_max_ value of ACh. The control E_max_ was 1.22 ± 0.11 g, while in the presence of carvacrol 100 μM, it was 1.09 ± 0.12 g (*p* = 0.73), and after washing, 0.98 ± 0.13 g (*p* = 0.93) (Figure 3b).

**Figure 4:**
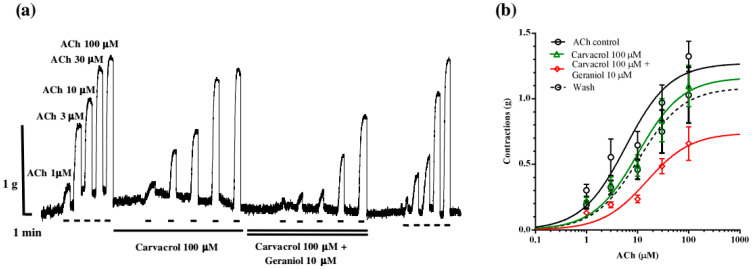
(**a**) Original recording of isometric contractions of *A. suum* muscle flap induced by increasing acetylcholine concentrations and the effect of carvacrol and carvacrol + geraniol (on those contractions); (**b**) the concentration–response plot for acetylcholine control, in the presence of carvacrol 100 μM in the presence of combination of carvacrol and geraniol 10 μM and after washing (n = 6) (mean ± S.E.).

The interaction between carvacrol and geraniol was tested on the contractions of the neuromuscular preparation of the parasitic nematode *Ascaris suum* in the same conditions as the previous examination of their individual effects (Figure 4a). The obtained control EC_50_ of ACh was 5.89 ± 1.45 μM. Incubation of neuromuscular preparations with carvacrol 100 μM non-significantly increased the EC_50_ to 10.10 ± 1.51 μM (*p* = 0.11). Furthermore, the addition of geraniol 10 μM in the presence of carvacrol increased the EC_50_ value of ACh significantly to 15.03 ± 1.52 μM (*p* < 0.0001). After removing carvacrol and geraniol from the pharmacological bath, the EC_50_ of acetylcholine was 10.64 ± 1.64 μM (Figure 4b). The maximal contractile effect of ACh in the control series was 1.27 ± 0.12 g, and it did not significantly change after incubation with carvacrol (1.24 ± 0.21 g). However, when the preparation was incubated with geraniol (10 μM) together with carvacrol, E_max_ decreased significantly (*p* = 0.0272) to 0.77 ± 0.14 g.

### 2.3. Effect of Geraniol and Carvacrol on Ascaris suum nAChR Expressed in Xenopus Oocytes

**Figure 5:**
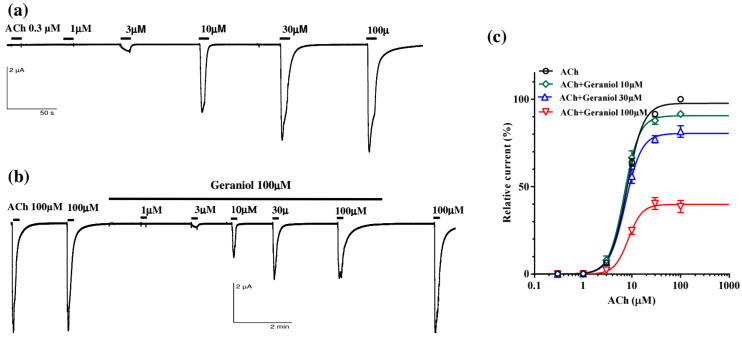
Geraniol effect on the acetylcholine concentration–response relationships for the *Ascaris suum* ACR-16 nAChR expressed in Xenopus oocytes; (**a**) representative ACh-evoked currents; (**b**) ACh-evoked currents in the presence of geraniol 100 µM; (**c**) concentration–response curves for ACh control and in the presence of geraniol at 10, 30 and 100 µM. All responses are normalized to ACh 100 µM. Results are shown as the mean ± S.E.

Considering the recorded interaction of carvacrol and geraniol on the contractions of the *A. suum* neuromuscular preparation, we examined their individual and joint effects on the homomeric *A. suum* nAChR (Asu-ACR-16) expressed in X. leavis oocytes. Perfusion of increasing concentrations of acetylcholine (ACh) caused a concentration-dependent increase in current, with a control EC_50_ value of 7.89 ± 1.02 μM and large currents with maximum amplitude in the μA range (Figure 5a).

We tested the effect of 10, 30 and 100 μM (Figure 5b) of geraniol on the current induced by increasing concentrations of ACh. We found that the ACh EC_50_ values were 6.95 ± 1.02 μM, 7.33 ± 1.02 μM and 8.44 ± 1.08 μM in the presence of 10, 30 and 100 μM of geraniol, respectively. These values were not significantly different compared to the control. The ACh-evoked maximal response amplitude of current (E_max_) in the control series was 97.63 ± 0.92%, and 10 μM of geraniol did not change this value significantly (90.53 ± 1.46%, *p* = 0.0803). However, higher concentrations, 30 and 100 μM of geraniol, significantly decreased the E_max_ to 80.41 ± 1.62% and 39.83 ± 1.49% (*p* < 0.0001; *p* < 0.0001), respectively (Figure 5c), indicating a non-competitive inhibition of ACh-elicited currents by geraniol.

**Figure 6:**
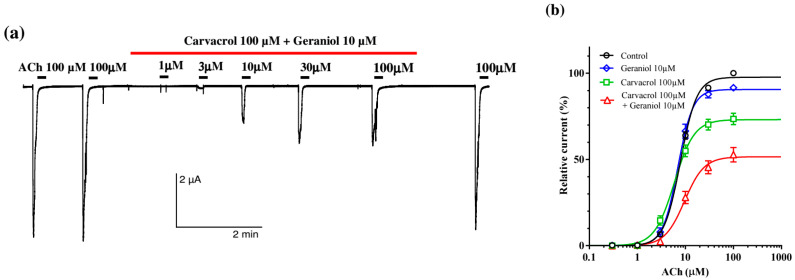
Effect of geraniol and carvacrol on the acetylcholine concentration–response relationships of *Ascaris suum* ACR-16 nAChR expressed in Xenopus oocytes; (**a**) representative ACh-evoked currents in the absence and presence of carvacrol 100 µM and geraniol 10 µM; (**b**) concentration–response curves for acetylcholine control (black line), and in the presence of geraniol 10 µM (blue line), carvacrol 100 µM (green line), geraniol 10 µM and carvacrol 100 µM (red line). All responses are normalized to ACh 100 µM. Results are shown as the mean ± S.E.

In the next series of experiments, we tested the effect of carvacrol and combination of geraniol and carvacrol on the ACh-evoked currents. Individually and in combination, carvacrol 100 μM and geraniol 10 μM did not significantly affect the value of EC_50_ of ACh. The control value was 7.89 ± 1.02 μM, 5.89 ± 1.07 μM in the presence of carvacrol, and 6.95 ± 1.04 μM for geraniol. Furthermore, the combination of geraniol and carvacrol insignificantly increased the EC_50_ value to 9.57 ± 1.11 μM (Figure 6a). However, the effect of the combination of carvacrol and geraniol on E_max_ was different. Carvacrol 100 μM significantly reduced E_max_ to 73.09 ± 1.87%, as we previously showed. Geraniol 10 μM by itself did not significantly affect E_max_, but in combination with carvacrol, it reduced E_max_ by half, i.e., to 51.54 ± 2.46% (Figure 6b).

## 3. Discussion

Molecular docking analysis in our previously published research indicated potential differences in the binding of carvacrol and geraniol to ACR-16, a homomeric nAChR widely distributed in Ascaris tissues [5]. Carvacrol shows affinity for an allosteric binding site in the beta domain (a possible allosteric site composed of two sub-sites located close to each other), while geraniol potentially binds to the receptor at two sites in the alpha domain with lower affinity than carvacrol. The presence of geraniol or the presence of carvacrol enhances their binding to the receptor. We also previously published information that carvacrol dominantly exhibited characteristics of a non-competitive antagonist of nAChR in *A. suum* [15,16], so we considered it important to analyze the influence of geraniol on the effect of carvacrol. The prediction of molecular interaction is verified by examining the effects of carvacrol and geraniol on the motility and survival of adult *C. elegans*. In our study, geraniol showed better efficacy but did not show a distinct time-dependent effect; on the other hand, carvacrol showed a slightly weaker efficacy but a clear time-dependent effect. However, when adult *C. elegans* was exposed to the combination of geraniol and carvacrol, the LC_50_ value after 24 h was reduced almost 10-times, and the effect was time dependent. The prediction from the docking that the presence of one ligand increases the binding of the other ligand was confirmed. It is also interesting that geraniol caused atonic paralysis, but before paralysis, it caused the cessation of pharyngeal pumping. Further investigation of this potential new target site for AI is of undoubted importance. These results are in agreement with the data that geraniol exhibits nematocidal effects and, at a concentration of 2%, reduces the motility of L3s of H. contortus, T. axei and T. circumcincincta by 82, 90 and 94% [17].

We checked the prediction about the synergistic interaction of geraniol and carvacrol in the model of contractions of the neuromuscular preparation of *A. suum*. The tested concentrations of geraniol (10 and 30 μM) decreased in the EC_50_ value of ACh (for 18.68%) as well as an increase in contractile E_max_ (for 4.43%) but without statistical significance. On the other hand, carvacrol at a concentration of 100 μM insignificantly increases the EC_50_ of ACh and decreases the E_max_ of contractions, which is in agreement with our previously published results [3]. However, when we incubated neuromuscular preparations of *A. suum* with the combination of carvacrol 100 μM and geraniol 10 μM, there was a significant increase in the value of EC_50_ of ACh and a significant decrease in the value for contractile E_max_. This corresponds to our results obtained with *C. elegans*, which indicate a significant synergistic interaction between carvacrol and geraniol against ACh-induced contractions.

To examine whether the interaction occurs at the receptor level, we tested the effects of carvacrol and geraniol on the homomeric Asu-ACR-16 expressed on X. leavis oocytes. In the presence of geraniol (30 and 100 μM), the ACh EC_50_ value remained unchanged, while the E_max_ was significantly reduced. This effect indicates a non-competitive antagonism or allosteric modulation caused by geraniol at Asu-ACR-16. Furthermore, we compared the effects of carvacrol (100 μM) and geraniol (10 μM) and their combination. As expected, carvacrol acted as a non-competitive antagonist on the *A. suum* N-AChR, as described previously [15,18], while the addition of geraniol reduced E_max_ significantly on almost 50% of the control value. This interaction is somewhat different from the interaction observed in the contraction tests. In both cases, the combined inhibitory effect in relation to ACh is greater than individual, but in contraction assays, in addition to the decrease in E_max_, the EC_50_ value of ACh increased. In tests on Asu-ACR-16, we obtained a synergistic inhibitory interaction of carvacrol and geraniol only in the reduction in E_max_, without changes in EC_50_. An explanation can be found in the fact that both antagonists bind to the allosteric site in Asu-ACR-16, resulting in non-competitive antagonism.

Asu-ACR-16 is a homopentameric nAChR with widespread distribution in the somatic muscle, pharynx, ovijector and head, which indicates various tissue-related functions. The *A. suum* channel is most sensitive to nicotine but insensitive to levamisole and pyrantel when compared with the same channel in *C. elegans* [19]. The difference in the interaction between contractile tests and electrophysiology experiments on expressed Asu-ACR-16 can be explained by the fact that carvacrol and geraniol in contraction assays can act on all types of nAChRs in the neuromuscular preparation of *Ascaris suum*. Here, we evidenced the effect on Asu-ACR-16, but we cannot rule out the possibility that carvacrol and geraniol could also act on other nAChR subtypes, including either UNC-29/UNC-38 channels [20] or ACR-26 channels [21], as well as additional nAChRs from *A. suum* that have not been characterized so far [22]. This hypothesis is supported by the significant effect of carvacrol previously reported on the morantel-sensitive nAChRs made of the ACR-26/ACR-27 subunits from *Parascaris* sp. [15]. On the other hand, it is interesting to comment on the greater efficacy of geraniol on *C. elegans*. This can be explained by differences in the antagonist pharmacology between the two ACR-16 homologues. The *A. suum* channel is indeed most sensitive to nicotine and insensitive to levamisole and pyrantel, as also observed with the *C. elegans* ACR-16 nAChR. Morantel behaved as a non-competitive antagonist of the *A. suum* nAChR but was less potent in comparison to its effect on the *C. elegans* receptor [23,24]. We will not comment more specifically, though it is tempting to speculate that *C. elegans* ACR-16 is more sensitive to geraniol as well.

## 4. Materials and Methods

### 4.1. C. elegans Testing

*C. elegans*, N2 wild-type, was obtained from the Caenorhabditis Genetics Center [25]. Worms were cultivated and adults were separated for testing, as previously explained in Stojković et al. [5]. Suspensions of adult nematodes (20 μL) were inoculated on the Petri dish (diameter 3 cm) with 2.5 mL of NGM substrate and increasing concentrations of carvacrol or geraniol (1, 3, 10, 30, 100, 300 ili 1000 µM) and a combination of geraniol and carvacrol 1:1 (1, 3, 10, 30, 100, 300 ili 1000 µM). The titer of adult worms was 20–37/20 μL, and each concentration was tested on three Petri dishes. The three Petri dishes without the added test substances were untreated controls.

Inoculated plates with *C. elegans* were placed at a temperature of 20 degrees Celsius for 24 or 48 h. After incubation, observation of worm motility, existence of pharyngeal pumping and checking of worm death were performed, as described previously [5].

### 4.2. Ascaris suum Contractions

Ascaris muscle strip for contraction studies was prepared, as previously described in Stojković et al. [5]. After 15 min of equilibration under a 500 mg load, contractions induced by increasing concentrations of acetylcholine (ACh) (1, 3, 10, 30 and 100 μM) were measured, as well as after incubation with geraniol, carvacrol or their combination. Monitoring, recording and expression of contraction intensity are described in Stojković et al. [5].

### 4.3. Electrophysiological Recordings

The functional reconstitution of the *A. suum* nicotine-sensitive acetylcholine receptors (nAChRs) was carried out in Xenopus laevis oocytes, as described previously [24]. Briefly, capped cRNAs encoding the *A. suum* ACR-16 subunit were synthesized in vitro using the mMessage mMachine T7 transcription kit (Thermofisher). Defolliculated Xenopus laevis oocytes (Ecocyte Bioscience) were micro-injected with 36 nL of *A. suum* ACR-16 cRNA at 50 ng/µL using the Nanoject II microinjector (Drummond) and incubated for 3 days at 19 °C to allow for nAChR expression. Two micro-electrode voltage-clamp experiments were performed using an Oocyte Clamp OC-725D amplifier (Warner Instruments) under voltage clamp at −60 mV, as previously described [5,15].

### 4.4. Drugs and Substances

Acetylcholine, geraniol and carvacrol were obtained from Sigma-Aldrich Co. (St Louis, MO, USA). Acetylcholine was dissolved in the APF-Ringer and Tyrode solution. Geraniol and carvacrol were dissolved in ethanol, with a final concentration of ethanol in the APF-Ringer and Tyrode Solution of 0.1%*v*/*v*.

### 4.5. Statistical Analyses

The results of the lethal effect of carvacrol and geraniol are presented in percentage (%), and the determinations of the median lethal concentration (LC_50_) were processed through nonlinear regression. The results of muscle contraction assay are expressed as means ± S.E. in grams (g) of contractions. The dose–response relationship was analyzed via nonlinear regression, and the values of the median effective concentration (EC_50_) of the agonist (ACh), without and in the presence of geraniol and carvacrol, were determined. Whole cell current electrophysiology responses were analyzed using the pCLAMP 10.4 package (Molecular Devices, San Jose, CA, USA). EC_50_ values were determined using nonlinear regression on normalized data (100 µM ACh as maximal response) using GraphPad Prism^®^ software. One-way analysis of variance (ANOVA) was applied for the comparison of the differences between the EC_50_ value and the maximal effect (E_max_). Differences were considered significant when the *p* value was <  0.05. The statistical analysis was conducted using GraphPad Prism^®^ software, Version 6.01 (San Diego, CA, USA), while all values are expressed as mean ± standard error (S.E.).

## 5. Conclusions

The presented research confirms the significant anthelmintic potential of the active ingredients of essential plant oils and the synergistic effect of their combinations. On the other hand, it is obvious that one of the important sites of synergistic anthelmintic interaction is the nematode nACh receptor. The possibility of the antiparasitic application of active ingredients of essential plant oils that exhibit a synergistic anthelmintic effect, considering the specific mechanism of action, may be an important platform for the development of new drugs and new therapeutic procedures.

## Data Availability

The data that support the findings of this study are incorporated into the article.
